# What if professional mosquito abatement in Africa started in a refugee camp?

**DOI:** 10.5281/zenodo.14278016

**Published:** 2024-12-04

**Authors:** Silas Majambere

**Affiliations:** 1 Chair, Board of Directors, Maison Shalom, KK Kicukiro, Kigali, Rwanda.

## Abstract

In the aftermath of the 2015 political crisis in Burundi, a humanitarian organisation, Maison Shalom, fled the country to Rwanda with tens of thousands of Burundians. In an attempt to assist their compatriots, a group of Burundians in the diaspora created the Académie *Ubuntu* and teamed with Maison Shalom to give online classes to the refugees. With courage and determination and despite the conditions in the refugee camp and the language barrier, 17 refugees successfully completed the ‘Best Practices for Integrated Mosquito Management Virtual Training Programme’, offered by the American Mosquito Control Association. These 17 refugees are determined to put these skills to work and perhaps start the very first mosquito abatement programme in Africa.

The country of Burundi, in East Africa, has experienced cyclical political violence since its independence. The most recent crisis, in 2015, resulted in hundreds of thousands of refugees fleeing to neighbouring countries, with Rwanda receiving approximately 60,000 refugees at the peak of the crisis.

Among the refugees was Maison Shalom, a non-governmental organisation that had been operating in Burundi since 1993. Its founder, Marguerite (Maggy) Barankitse, had become wanted by the government of Burundi for opposing violations of human rights during the 2015 crisis.

Against all odds, Maison Shalom, having lost everything they had built over twenty years in Burundi, managed to resume their work with the same passion to share love, protect lives and renew hope for fellow refugees, this time in Rwanda.

Among several programmes and activities at the Mahama refugee camp in Rwanda, Maison Shalom built a hall equipped with computers and free internet, providing refugees with the opportunity to educate themselves and stay connected with the world.

In 2018, six members of the Burundian diaspora – refugees from an earlier period who have since become citizens of their host countries in Europe and USA – deliberated on the best way to support their compatriots in the Mahama refugee camp. Leveraging the facilities that Maison Shalom was building for refugees in Rwanda, they decided to create ‘Académie Ubuntu’.

The concept of *Ubuntu,* loosely translated as ‘I am because we are’, is inspired by a pan-African ideology which emphasises that human lives are interdependent, and life only thrives in a community where every person is dignified. Académie Ubuntu aims to foster resilience and development through tailor-made training within the refugee community in Rwanda. The training uses physical and virtual conferences and workshops as tools for transmitting knowledge. In the spirit of Ubuntu, the beneficiaries of these trainings have the responsibility to voluntarily give back and share the values and knowledge they have acquired with their communities. This educational approach is aimed at young and adult refugees of all genders, social and cultural backgrounds, and at any level of experience and knowledge. The goal is to enhance the resilience, leadership and socio-economic environment of refugees, equipping them to participate in the life and development of the host country and, eventually, their country of origin when they return.

## Refugees and the science of mosquito control

The refugee camp in Mahama is situated in the district of Kirehe, one of the malaria hotspots in Rwanda. The camp is at the shores of the river Akagera which creates flooded areas during high tide and these water bodies offer ideal breeding grounds for mosquitoes, including malaria vectors. The government of Rwanda distributes bednets in the camp to prevent malaria; however, there is no sustained mosquito control programme in the area. This situation is not unique to this camp. Although mosquito-borne diseases such as malaria and dengue claim close to a million lives every year in Africa, to date there is no single country in Africa that has a functional mosquito abatement programme. The only attempts at controlling mosquitoes in Africa occur inside houses (using bednets and spraying houses with residual insecticides) and there is a complete lack of integrated mosquito management programmes. This approach is in direct contrast with countries outside Africa where professional mosquito abatement programmes largely focus on the outdoors and follow an integrated vector management strategy.

In October 2023, the American Mosquito Control Association (AMCA) launched the ‘Best Practices for Integrated Mosquito Management Virtual Training Program’, a comprehensive resource designed to ‘enhance skills, advance mosquito control programs, and elevate public health protection’ and a ‘gateway to mastering the science-based approach to mosquito control’ [1].

Given the risks posed by mosquitoes in Africa, and the heightened risk to vulnerable people such as refugees, Académie Ubuntu seized the opportunity offered by the AMCA programme and encouraged refugees to enrol. The course consisted of 13 modules delivered by 24 leading experts in mosquito control. To obtain certification in Integrated Mosquito Management, candidates had to pass a 100-question exam with a score of at least 80%. The challenge was significant because none of the enrolled candidates had prior training in mosquito control; their educational background was entirely in French, and since the programme was conducted in English, this constituted a language barrier. Despite this challenge, 21 candidates enrolled in February 2024 with a condition set by the organisers of the programme to complete the course by June 2024.

**Figure 1.**
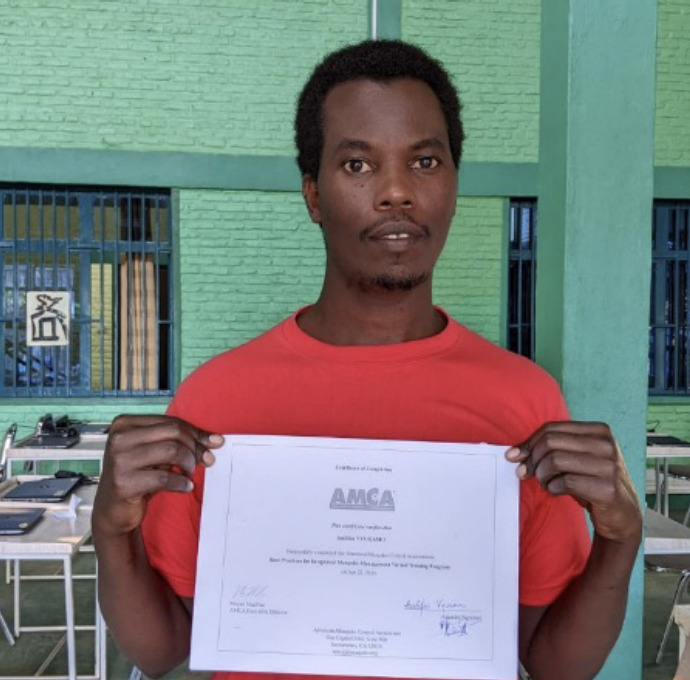
Audifax Vyugamo, the first candidate to finish the AMCA course on integrated mosquito management.

The role of the Académie Ubuntu trainer for this course was to support the enrolled candidates through the registration process, help them understand the aim of the course, and being available to clarify any complexities the candidates experienced. The trainer followed the candidates through Module 4 only but thereafter was unable to continue following their progress. By the time the trainer returned, one candidate had finished the entire programme and received certification (Figure 1). He had completed the course in just two months. Two weeks later, three more candidates had finished the programme, and before the deadline in June, 17 of the 21 registered candidates had completed all the modules and were certified.

## Lessons learnt and the way forward

One sentence that Maggy Barankitse, Founder of Maison Shalom, repeats often is that ‘refugees are normal people in an abnormal situation’. This truth could not be more evident. The fact that these candidates pursued this programme despite the challenges of life as a refugee is a testament to their resilience and sheer determination to succeed. Their instinctive motivation is survival and overcoming the despair that often plagues refugees who do not see hope in the future. Typically, this course is taken by already employed mosquito professionals who want to improve their skills or newcomers to the field of mosquito control who have a profession in sight. Not so for these candidates.

The refugees who enrolled in and completed this course demonstrate that even in desperate circumstances, when given a sign of hope and encouragement, human beings can overcome and thrive. While the world faces a complex immigration crisis - marked by thousands of Africans drowning in the mediterranean sea and others becoming pawns in the political elections in Europe and the USA - theses refugees in Rwanda demonstrate that they are normal humans with untapped talents and abilities that could be utilised to make the world a better place. The humane and welcoming policies of Rwanda as a host country, the amazing work Maison Shalom has been doing for the past thirty years, and the humble contribution of Académie Ubuntu all played a role in unveiling the abilities of these candidates.

Bolstered by this experience, Académie Ubuntu is determined to push this example of success to the next level. Building on the theoretical course they successfully completed, the 17 candidates will need to be trained in practical mosquito management. This will require time from trainers and some basic entomological equipment for the candidates. Upon completing the practical field training programme, the candidates intend to apply with the local authorities for certification as mosquito control professionals and to start a mosquito abatement programme in the refugee camp and nearby host communities. If supported and successful, this will constitute the very first mosquito abatement programme on the continent of Africa.

What if this long-awaited change in mosquito management came from a refugee camp in Mahama, Rwanda?
